# Relação entre Norepinefrina Urinária, Fibrose e Arritmias na Cardiopatia Chagásica Crônica com Fração de Ejeção Preservada ou Minimamente Reduzida

**DOI:** 10.36660/abc.20210400

**Published:** 2022-07-07

**Authors:** Eduardo Marinho Tassi, Emília Matos do Nascimento, Marcelo Abramoff Continentino, Basilio de Bragança Pereira, Roberto Coury Pedrosa

**Affiliations:** 1 Universidade Federal do Rio de Janeiro Faculdade de Medicina Instituto do Coração Edson Saad Rio de Janeiro RJ Brasil Instituto do Coração Edson Saad, Faculdade de Medicina, Hospital Universitário Clementino Fraga Filho, Universidade Federal do Rio de Janeiro, Rio de Janeiro, RJ – Brasil, Rio de Janeiro, RJ – Brasil

**Keywords:** Arritmias cardíacas, Fibrose miocárdica, Cardiomiopatia chagásica, Denervação Autonômica, Norepinefrina

## Abstract

**Fundamento:**

Na cardiomiopatia chagásica crônica (CCC), impõem-se estudos com a proposta de identificar fatores de risco arritmogênicos em pacientes nos quais a disfunção ventricular de moderada a grave não está presente.

**Objetivos:**

Verificar a dependência entre arritmias ventriculares frequentes (ARV), fração de ejeção de ventrículo esquerdo (FEVE), extensão da fibrose pela ressonância magnética cardíaca (RMC) e dosagem de norepinefrina urinária (NOREPI) na CCC com FEVE preservada ou minimamente comprometida.

**Métodos:**

Foi analisada no Holter a presença de extrassístoles ventriculares >30/hora. Na RMC, avaliou-se a FEVE e a quantificação de massa fibrosada. Foi realizada a dosagem de NOREPI pelo método de Muskiet. A matriz de correlação foi calculada para aferir a capacidade de as variáveis preverem outra sendo considerado significante p<0,05.

**Resultados:**

Foram incluídos no estudo 59 pacientes, com idade média de 57,9±10,94 anos. Arritmia ventricular frequente (ARV) foi detectada em 28 pacientes. A variável fibrose mostrou-se inversamente proporcional à fração de ejeção de ventrículo esquerdo (FEVE) (R de −0,61) e à norepinefrina urinária (NOREPI) (R de −0,68), assim como a variável ARV mostrou-se inversamente proporcional à FEVE (R de −0,33) e à NOREPI (R de −0,27). Já a FEVE mostrou-se diretamente proporcional à NOREPI (R de 0,83).

**Conclusão:**

Nesta amostra, em pacientes com CCC com FEVE preservada ou discretamente reduzida, observa-se a integridade do sistema nervoso autonômico em corações com pouca fibrose e FEVE mais elevada, apesar da presença de tradicionais fatores de risco para morte súbita cardíaca. Há dependência entre os níveis de NOREPI, FEVE e fibrose miocárdica, mas não com ARV.

## Introdução

A doença de Chagas (DC) ainda apresenta marcada importância epidemiológica em razão do contingente de infectados com potencial para desenvolvimento de formas graves. Estima-se que, no Brasil, haja 1,2 milhão de pessoas acometidas^1^ pela patologia, um terço delas cardiopatas, das quais dois terços com o coração minimamente comprometido.^2^

A cardiomiopatia chagásica crônica (CCC) é considerada uma condição arritmogênica decorrente de uma miríade variável de arritmias potencialmente fatais, principalmente em estágios avançados da doença (grupo com alto risco individual de morte súbita elétrica cardíaca).^3,4^ Embora os pacientes de alto risco de morte súbita elétrica possam ser identificados por seus fatores de risco, na população com DC, o maior número de casos de morte súbita elétrica é registrado em pacientes não previamente determinados como de alto risco.^3^ Esse aparente paradoxo dificulta a adoção de medidas preventivas em larga escala e justifica estudos nesse grupo de pacientes com fração de ejeção preservada ou discretamente reduzida, ainda que seja motivo de debate.^3,4^

O mecanismo que envolve a gênese das arritmias ventriculares nos estágios precoces da CCC pode estar relacionado com a denervação autonômica, característica marcante da DC.^4–6^ Estudos da última década^7–9^ demonstraram que a denervação autonômica cardíaca (com diferentes níveis de gravidade) é achado comum em pacientes com DC e ocasionado por inflamação neuronal e ganglionar. A destruição com perda variável de células nervosas inicia-se na fase aguda e continua na fase crônica, provocada por mecanismos imunes ou parasitários, agindo exclusivamente ou em combinação.^10,11^ A denervação autonômica tem importância no entendimento da patogênese, assim como na história natural da CCC.

A fim de caracterizar os níveis de norepinefrina como um indicador da atividade simpática, muitos autores^12–14^ já demonstraram a relação direta entre estimulação neural simpática e norepinefrina.

Em pacientes com ICC, a hiperatividade simpática é persistente e relacionada aos sintomas clássicos de estímulo adrenérgico, tais como taquicardia, sudorese fria, diarreia e ansiedade. Porém, na cardiopatia chagásica, esses aspectos permanecem controversos, havendo indicadores de que o sistema simpático caminha para a exaustão à medida que avança o comprometimento cardíaco.

O envolvimento comum de áreas distais do miocárdio de ventrículo esquerdo (VE), como o ápice, e de segmentos ínfero-laterais basais, sugere uma inflamação aguda, levando à isquemia miocárdica decorrente da desregulação microvascular na patogênese da fibrose miocárdica desses pacientes. Corroborando isso, a regulação do fluxo anormal microvascular na presença de inflamação miocárdica crônica na CCC foi demonstrada por cintilografia^15–17^ e ressonância magnética.^18^ Essas anormalidades de perfusão geralmente precedem o aparecimento de alteração segmentar, sugerindo que distúrbios microvasculares podem se desenvolver antes do início de dano miocárdico e ser um agente causador de fibrose miocárdica.

A relação entre os achados da RMC e as arritmias na CCC já foi avaliada por muitos estudos e atualmente é recomendada em caso de pacientes com arritmia ventricular grave para quantificar a extensão de fibrose miocárdica e risco de morte súbita.^19^

Assim, o grupo populacional portador de DC com potencial para desenvolver complicação cardíaca é suficientemente grande para justificar estratégias diagnósticas que identifiquem grupos de risco.^20^ Portanto, o objetivo deste trabalho é verificar a dependência entre as variáveis arritmia ventricular frequente, extensão da fibrose, função ventricular, alteração segmentar e dosagem de norepinefrina urinária em pacientes com CCC.

## Métodos

Foram incluídos os pacientes com CCC com idade superior a 21 anos e função ventricular esquerda preservada ou minimamente comprometida (FE >45%) à RMC e com dosagem de norepinefrina urinária anterior à data da RMC, a qual foi realizada entre março e dezembro de 2010, e precedida de eletrocardiograma (ECG) e Holter de 24 horas. Somente foram incluídos pacientes assintomáticos afastados de zona endêmica há mais de 20 anos e em uso de betabloqueadores e inibidores de enzima de conversão de angiotensina (IECA). Foram excluídos pacientes que apresentassem disfunção renal (*clearance* estimado de creatinina <30 mL/min), passado de ablação por estudo eletrofisiológico, diabetes ou mais que dois fatores de risco para doença coronária, fibrilação atrial, tempo de eco (TE) compatível com isquemia miocárdica, infarto do miocárdio prévio, qualquer procedimento de revascularização miocárdica ou periférica ou contraindicação para ressonância magnética cardíaca (marca-passo definitivo, cardiodesfibrilador implantado, clipe neurocirúrgico ou implante coclear).

Arritmia ventricular frequente foi considerada no Holter pela presença de extrassístoles ventriculares >30/hora ou episódios de taquicardia ventricular não sustentada (TVNS) (definida como três ou mais batimentos consecutivos com duração <30 segundos).^21^

A dosagem de norepinefrina urinária foi aferida ao longo dos anos de 2004 a 2006. Todos os pacientes foram orientados a evitar a ingestão de alimentos que contêm tiramina (substância que facilita a liberação da norepinefrina dos locais de armazenamento (no interior dos neurônios)), capazes de interferir na concentração de norepinefrina pelo menos 24 horas antes e durante o período de armazenamento da urina. O uso de betabloqueadores não foi suspenso durante a coleta. A coleta de urina, procedimento feito por 24 horas, teve início num domingo, às 6h e término na manhã de segunda-feira. Todas as amostras foram armazenadas em dois frascos de polietileno com capacidade de um litro cada um, contendo cada frasco 1 ml de HCl 6 M (pH 1.0), com a recomendação de que as amostras fossem mantidas a 4°C durante o período de coleta (24 horas). O método utilizado para determinação da norepinefrina urinária teve como base a proposição de Muskiet et al.^22^

A RMC foi realizada em um equipamento GE HDX de 1,5 Tesla (Wakeusha, Wisconsin), sendo adquiridas duas sequências de pulso: a primeira foi cine-RMC (Steady-State Free Precession − SSFP) em eixo longo e eixo curto para mensuração e cálculo de massa, volumes e FEVE. O corte mais basal no eixo curto foi posicionado logo após o anel atrioventricular, e todas as subsequentes pausas respiratórias em expiração máxima foram adquiridas com 8 mm de espessura e espaçamento de 2 mm entre os demais cortes, até o ápice de VE. Os parâmetros utilizados foram FOV (sigla para a expressão inglesa *field of view*) 400 mm, matriz 224 × 224, 20–24 linhas/segmento, resolução temporal <50 ms, tempo de repetição (TR) = 3,9 ms, tempo de eco (TE) = 1,5 ms, *flip angle* de 50° e número de excitações (NEX) de 1. Após 3 minutos da injeção de 0,3 mmol/kg de gadolínio (Dotarem®, Guerbet), uma segunda sequência realizada foi gradiente eco com recuperação de inversão (técnica do realce tardio) em eixo longo e eixo curto, para pesquisar fibrose miocárdica com os seguintes parâmetros: FOV 360 mm, matriz 224 × 192, 24 linhas/segmento, TE = 2,9 ms, *flip angle* de 20°, espessura de corte de 8 mm com espaçamento de 2 mm e NEX de 2.

A mensuração e os cálculos de medidas de VE e VD foram realizados independentemente por dois investigadores cegos sobre a qual grupo os pacientes pertenciam, em estação de trabalho dedicada a exames cardiológicos por RMC, por meio de *software* específico (Report CARD®, versão 3.6, GE).

O cálculo da massa fibrosada, se presente, foi realizado por meio de aplicativo específico do *software* mediante a detecção semiquantitativa de áreas hiperintensas compatíveis com a fibrose nas sequências de realce tardio em eixo curto, em que o investigador tinha a liberdade de editar os limites da área de fibrose.

Este projeto de estudo foi aprovado pela Comissão de Ética em Pesquisa do Hospital Universitário Clementino Fraga Filho da Universidade Federal do Rio de Janeiro, atendendo às diretrizes nacionais e internacionais para pesquisa em seres humanos (Resolução n° 466/2012 do Conselho Nacional de Saúde), que regulamentam experimentos envolvendo pessoas.

### Análise Estatística

Com base em estudos prévios, foram utilizadas as variáveis já conhecidas de fatores de risco para instabilidade elétrica: >30 extrassístoles por hora,^21,23^ idade,^24^ alteração segmentar,^24,25^ FEVE e fibrose miocárdica.^25–27^ Além dessas, foi acrescentado o nível de norepinefrina urinária.

A normalidade dos dados foi verificada pelo teste de Shapiro-Wilk juntamente com o *boxplot* e o gráfico quartil-quartil. As variáveis normalmente distribuídas foram expressas pela média±desvio-padrão e as que não seguem a distribuição normal, pela mediana e intervalo interquartil.

Para análise de arritmia, foi utilizado ponto de corte de 720 extrassístoles em 24 horas ou presença de TVNS^(21)^. Já a alteração segmentar foi avaliada pela presença ou ausência pela RMC (categórica). Para definir os pontos de corte das variáveis FEVE, fibrose miocárdica, nível de norepinefrina urinária e idade, foram elaboradas árvores de regressão usando-se como desfecho a arritmia.

Na sequência, com o ponto de corte já estabelecido, um modelo log-linear foi utilizado para mensurar as dependências das variáveis acima descritas e para confirmar os resultados obtidos por meio da árvore de regressão. As arestas de cada gráfico apresentam a medida de dependência das variáveis discretas e um número chamado Cramér's V, o qual consiste num algarismo entre 0 e 1, que indica quão fortemente duas variáveis categóricas estão associadas. Aqui cabe uma rápida explicação estatística: se quisermos saber se duas variáveis categóricas estão associadas, nossa primeira opção é o teste de independência do qui-quadrado. Um valor-p próximo de zero significa que é improvável que nossas variáveis sejam completamente desassociadas em alguma população. No entanto, isso não significa que as variáveis estejam fortemente associadas; uma medida que indica a força da associação é o Cramér's V.

Depois, foi realizada a matriz de coeficiente de correlação para aferir a capacidade de uma variável contínua prever outra, analisando-se idade, FEVE, fibrose, arritmia e norepinefrina urinária. O *software* R foi utilizado para a análise dos dados. Foi considerado significante valor de p<0,05.

## Resultados

Do grupo de 328 pacientes do ambulatório, 61 (23 masculinos) preencheram os critérios para participar do estudo. Dois não realizaram a fase pós-contraste da RMC (realce tardio), um por dificuldade de acesso venoso e outro pela história de atopia ao gadolínio, e foram excluídos posteriormente.

Os principais dados da amostra estão na Tabela 1, trata-se de pacientes cardiopatas crônicos com fração de ejeção normal ou discretamente diminuída. A carga fibrótica cardíaca (média de 15 g) esteve presente em praticamente metade dos pacientes e arritmias ventriculares significativas em 47% deles. Os níveis de norepinefrina urinária foram variáveis. Na Tabela 2, encontram-se os valores de corte obtidos pelas árvores de regressão linear.

**Tabela 1:** Dados gerais

Idade		
	Média±DP	57,9±10,9
**IMC**		
	Média±DP	26,1±4,8
**Gênero**		
	Feminino	36
	Masculino	23
**ECG**		
	Alt. repolarização	33
	HBASE	5
	BRD	1
	BRE	1
	BRD + HBASE	18
	BAV 1° grau	1
**Alteração segmentar**		
	Sim	19
	Não	40
**FEVE**		
	45–50%	7
	>50%	52
	Média±DP (%)	66,8 ±11,9
**Fibrose (realce tardio)**		
	Presente	27
	Ausente	32
	Mediana (IQ) (g)	0 (0; 10,9)
**Holter**		
	Sem arritmia	12
	Entre 1 e 719 ESV	19
	>720 ESV	28
	Mediana (IQ)	489,0 (3,0; 1813,5)
**Norepinefrina (nmol/24h)**		
	Mediana (IQ)	2369,6 (2233,6; 2502,1)
	Sem arritmia (IQ)	2429,1 (2334,5; 2497,6)
	Com arritmia (IQ)	2364,1 (2180,1; 2512,3)
	Sem fibrose (IQ)	2437,1 (2342,9; 2759,7)
	Com fibrose (IQ)	2327,4 (1461,1; 2429,1)

IMC: índice de massa corporal; ECG: eletrocardiograma; HBASE: hemibloqueio anterossuperior-esquerdo; BRD: bloqueio de ramo direito; BRE: bloqueio de ramo esquerdo; BAV: bloqueio atrioventricular; FEVE: fração de ejeção de ventrículo esquerdo; ESV: extrassístole ventricular; IQ: interquartil.

**Tabela 2:** Resultados da árvore de regressão linear para os pontos de corte para o log-linear

FEVE (n)	Alteração segmentar (n)	Arritmia (n)	Fibrose (n)	Norepinefrina (n)	Idade (n)
≤57% (13)	Não (41)	Não (31)	≤10,56% (44)	≤2218,97nmol/24h (15)	≤54 anos (20)
>57% (46)	Sim (18)	Sim (28)	>10,56% (15)	>2218,97nmol/24h (44)	>54 anos (39)

FEVE: fração de ejeção de ventrículo esquerdo.

**Figura 1:**
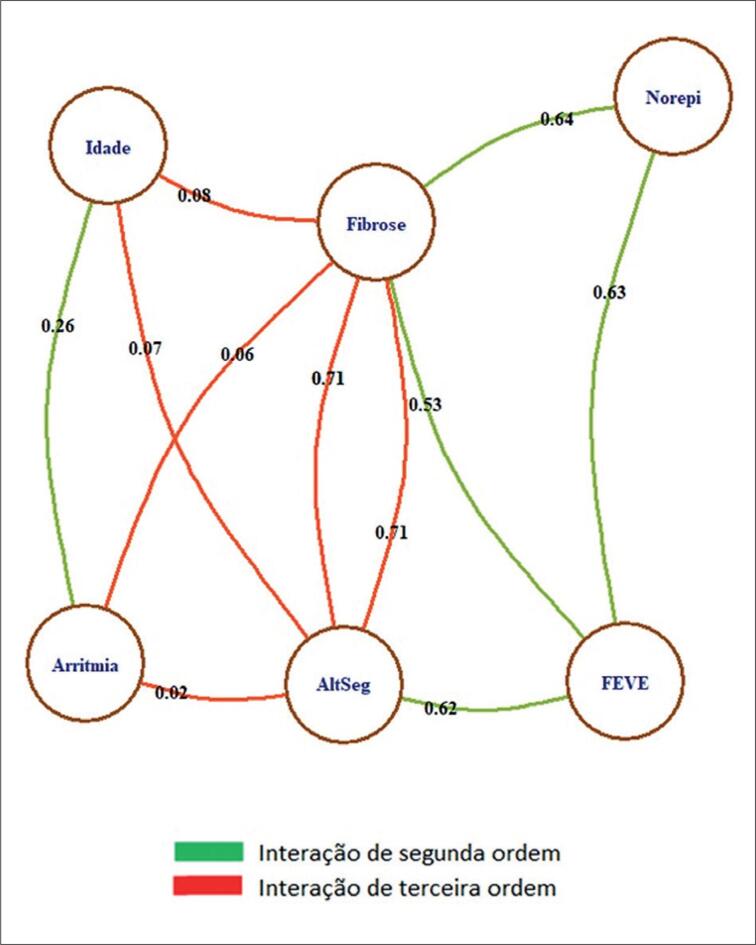
Modelo Log-Linear. Os pesos nas arestas correspondem à estatística Cramér's V (medida de dependência entre as variáveis discretas).

Em análise multivariada, por meio do modelo log-linear, verificou-se o padrão de interação (dependência) das variáveis demonstradas na Figura 1.

Observa-se que as variáveis fibrose, FEVE e norepinefrina apresentam padrão direto de interação (dependência) entre si, com alto poder de associação (fibrose e norepinefrina 0,64, FEVE e norepinefrina 0,63 e fibrose e FEVE 0,53). Essas interações são de segunda ordem. A fibrose está relacionada à arritmia na dependência de alterações segmentares por uma interação de terceira ordem, ou seja, as três variáveis têm que estar presentes. Observa-se também que não há interação direta de arritmia e norepinefrina.

**Figura 2:**
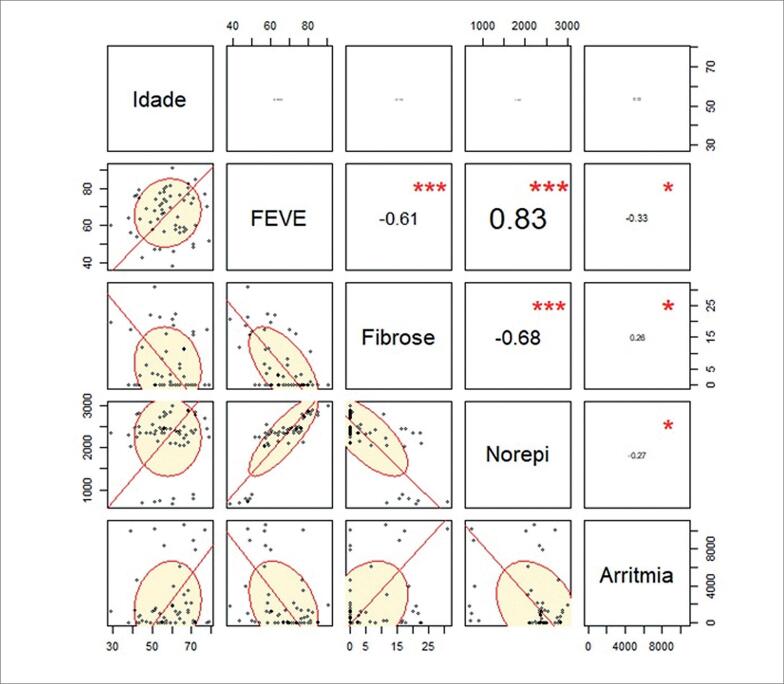
Matriz de Correlação (valores em R). Quanto mais ovalado melhor é a correlação. Os asteriscos representam a significância de acordo com o p-value. (*** p < 0.001, ** p < 0.1, * p < 0.05).

Em seguida, foi aplicada a matriz de correlação, em que R demonstra em percentual o quanto as cinco variáveis estão correlacionadas entre si (Figura 2). As variáveis com maior relação (diretamente ou inversamente proporcional) se encontram com a circunferência mais ovalada. Os asteriscos representam o nível de significância de acordo com o valor-p (*** p<0.001, ** p<0.1, * p<0.05). A variável fibrose mostrou-se inversamente proporcional à FEVE encontrada (R de −0,61) e à norepinefrina urinária (R de −0,68). Assim como a variável arritmia mostrou-se com relação inversa à FEVE (R de −0,33) e à norepinefrina urinária (R de −0,27). Já a FEVE mostrou-se diretamente proporcional à norepinefrina (R de 0,83).

## Discussão

Este é o primeiro estudo de CCC, com coração estruturalmente preservado com pouca fibrose e FEVE preservada ou minimamente alterada, a mostrar dependência significativa entre os fatores de risco tradicionais, pois pacientes com mais fibrose apresentam níveis de norepinefrina mais baixos e, quanto maior a FEVE, maiores os níveis de norepinefrina, como demonstrado na matriz de correlação com excelente associação (-0,68 e 0,83, respectivamente). Também foi evidenciado que a presença de arritmia não tem interação significativa com as demais variáveis.

Esta publicação é original ao mostrar pela primeira vez que mesmo em pacientes com pouca ou nenhuma disfunção ventricular, fatores de risco para morte súbita podem estar presentes, tais como denervação simpática (demonstrada pelos níveis de norepinefrina urinária), fibrose miocárdica e arritmias ventriculares frequentes. Ademais, traz mais relevância ao lembrar que a maior parte dos pacientes com DC estão no grupo com nenhuma ou discreta disfunção ventricular.

A dependência dos valores de norepinefrina mais elevados com maior FEVE, por meio da matriz de correlação neste trabalho, foi mostrado também com Iosa et al.^28^ mas com outro desenho de estudo, no qual se demonstrou a relação inversa da disfunção cardíaca na CCC com os níveis de norepinefrina ao evidenciar que nas fases tardias da cardioneuropatia chagásica os níveis plasmáticos de norepinefrina permaneceram normais, diferentemente dos pacientes com insuficiência cardíaca não chagásica, os quais apresentavam nível de norepinefrina mais elevado quanto maior a disfunção ventricular.

As evidências de lesão ao SNA já demonstradas em estudos experimentais na fase aguda e crônica da DC justificam as dependências entre os níveis de norepinefrina, fibrose e FEVE observadas neste estudo. Esses pacientes parecem apresentar denervação simpática causada por progressiva destruição neuronal refletida pela relação inversa entre os níveis de norepinefrina e fibrose miocárdica, como mostrada na matriz de correlação (Figura 2).

É sabida a alta variabilidade nos níveis de catecolaminas durante o ciclo circadiano ou até pela punção venosa para coleta da norepinefrina plasmática^29^ ou se o paciente se encontra internado ou não.^30^ Grande parte dos resultados de análise de norepinefrina publicados são baseados em amostras de plasma, e poucos estudos foram em DC. Ross et al.^31^ demonstraram que a determinação de norepinefrina em urina de 24 horas reduz o índice de resultados falso-negativos em pacientes com feocromocitoma.

Foi encontrado apenas um estudo clínico utilizando norepinefrina urinária na DC. Cunha et al.^32^ avaliaram o envolvimento do SNA na patogênese da CCC e evidenciaram diminuição dos níveis de norepinefrina urinária em CCC com disfunção ventricular e, de maneira inversa, níveis normais ou até aumentados em pacientes na forma indeterminada da DC.

Como demonstrado pelo nosso grupo anteriormente,^25^ a fibrose tem relação inversa com a FEVE e, neste estudo, ficam nítidas, pelo log-linear, as interações de segunda ordem (dependência direta) da fibrose, alteração segmentar e norepinefrina com a FEVE, o que em conjunto ou individualmente provoca uma remodelação ventricular, justificando em parte o modelo de Myerburg.^33^ No estudo anterior, havia uma interação de terceira ordem (as três variáveis têm que estar presentes) entre fibrose, arritmia e FEVE, a qual não se manteve no estudo atual. Isso pode ser explicado por dois motivos: em primeiro lugar, já que morfologicamente a denervação simpática ocorre antes da instalação da fibrose, isto pode equilibrar a dependência da arritmia perdendo poder explicativo.^9,34^ O segundo motivo é de desenho, pois no primeiro estudo, o log-linear somente levou em consideração os pacientes com FEVE acima de 50% pela RMC, ou seja, retirando-se sete pacientes com disfunção discreta (FEVE entre 45–50%), dos quais seis tinham arritmias frequentes, justifica-se a perda de interação das variáveis.

Em nosso estudo, a média de gramatura de fibrose miocárdica foi de 15,02 g (Tabela 1), em que o valor de 10,56% (10,01 g) determinou a presença de arritmias frequentes, de acordo com a árvore de regressão. Recentemente, Senra et al.^26^ observaram em um estudo prognóstico retrospectivo que a fibrose miocárdica é um preditor independente de risco (morte, disparo de cardiodesfibrilador implantável e transplante cardíaco) na CCC e que, a cada 1 g adicional de fibrose, aumentaria em 3,1% o risco de evento duro. Eles detectaram gramatura média de fibrose (15,2 g, ou 13,5%) em quantidade muito próxima à encontrada em nosso estudo, e pela curva ROC, determinaram que o ponto de corte de 12,3 g é um preditor de evento maior. No mesmo ano, Volpe et al.^27^ em outro estudo prognóstico retrospectivo de cerca de três anos de acompanhamento de pacientes com CCC, detectaram 10,4 g de fibrose (9,2% de massa de VE) e 11 óbitos — 10 desses pacientes tinham fibrose detectável.

Gadioli et al.^9^ encontraram relação proporcional entre a gravidade da arritmia (TVS e TVNS) com a extensão da denervação simpática de forma semelhante, a qual foi evidenciada neste estudo pela presença de arritmia com níveis mais baixos de norepinefrina, denotando maior denervação. Contudo, eles não encontraram relação entre fibrose detectada por ^99m^Tc-Sestamibi com arritmia ventricular, o que ocorreu neste estudo. Isso pode ser explicado pela maior resolução da RMC para detectar fibrose miocárdica mediante cintilografia miocárdica.

Este estudo apresenta algumas limitações. A definição de arritmia ventricular frequente no presente estudo pode ser questionável do ponto de vista clínico, porém não funcional em termos de modulação neurogênica. Contudo, ao utilizar uma população de DC de baixo risco (FEVE >45% e idade média de 57,9 anos) e focando na fundamentação anatomopatológica do substrato arritmogênico da fibrose e não na instabilidade clínica por arritmia maligna, confirma-se que o substrato arritmogênico já se encontra presente em tal população. Da mesma forma, evidencia-se neste estudo que o mecanismo patogênico predominante nesta população em questão é neurogênico e não cardíaco, fato também já publicado por outros autores.^8,35^

Embora não possamos descartar definitivamente o diagnóstico de doença cardíaca coronária como um importante fator de confusão, dados clínicos e padrão de fibrose distinto de doença isquêmica à RMC permitem inferir a ausência de doença coronariana obstrutiva funcionalmente significativa. Ademais, esse grupo de pacientes, invariavelmente, não apresentava indicações para coronariografia.^36^

A latência entre a dosagem de norepinefrina urinária e a RMC (cerca de seis anos) pode parecer grande, contudo é importante lembrar que a progressão de pacientes nos grupos de estágios iniciais de comprometimento cardíaco é muito lenta, cerca de 1,48 caso a cada cem pacientes/ano,^37^ ou seja, talvez, no máximo, seis pacientes tenham seu grupo alterado ao longo do estudo.^38^

Os pacientes faziam uso prévio (minimo de seis meses) de betabloqueadores e inibidores da enzima conversora de angiotensina. Todos utilizaram as drogas para controlar as complicações, de acordo com recomendações da literatura para DC^39^ na forma cardíaca (estádios A e B1). A decisão de tratamento farmacológico foi dentro do contexto clínico e não para controle ou tratamento de arritmias. Portanto, mesmo que modulem a resposta neurohormonal, essas drogas não seriam uma limitação do estudo, uma vez que a carga adrenérgica foi estabelecida na vigência do uso crônico desses fármacos.

O modelo log-linear mostra que a quantificação da arritmia não tem relação com a FEVE, fibrose e/ou norepinefrina, e a fibrose tem relação com norepinefrina urinária e FEVE, com poder de explicação semelhante entre si (estatística de Cramér's V de 0,53, 0,63 e 0,64). Realmente, há dados na literatura sobre DC indicando que a arritmia ventricular e/ou bloqueio de ramo direito (BRD) não são marcadores prognósticos independentes para morte em geral, contudo são marcadores de envolvimento cardíaco.^21^ O mecanismo de morte súbita na DC é por taquicardia ou fibrilação ventricular não necessariamente precedidas de arritmias complexas, estas estão mais relacionadas com a disfunção de VE.^4^

## Conclusões

Nos pacientes com CCC com fração de ejeção preservada ou discretamente reduzida, observa-se a integridade do SNA em corações com pouca fibrose e FEVE mais elevada, apesar dos tradicionais fatores de risco para morte súbita cardíaca. Há dependência entre os níveis de norepinefrina urinária, FEVE e fibrose miocárdica, mas não com arritmias ventriculares frequentes.
