# Supplementary material for: Relação entre Norepinefrina Urinária, Fibrose e Arritmias na Cardiopatia Chagásica Crônica com Fração de Ejeção Preservada ou Minimamente Reduzida
Source: Arq Bras Cardiol. 2022 Jul 7;119(1):3–11. [Article in Portuguese] doi: 10.36660/abc.20210400 (PMC9352138; doi:10.36660/abc.20210400)
Supplement: Translation

# Translation: Relationship between Urinary Norepinephrine, Fibrosis, and Arrhythmias in Chronic Chagas Heart Disease with Preserved or Mildly Reduced Ejection Fraction

**Authors:** Eduardo Marinho Tassi, Emília Matos do Nascimento, Marcelo Abramoff Continentino, Basilio de Bragança Pereira, Roberto Coury Pedrosa

**Affiliations:** 1 Rio de Janeiro RJ Brazil Instituto do Coração Edson Saad, Faculdade de Medicina, Hospital Universitário Clementino Fraga Filho, Universidade Federal do Rio de Janeiro, Rio de Janeiro, RJ – Brasil,^1^ Rio de Janeiro, RJ – Brazil, Rio de Janeiro, RJ – Brazil

**Keywords:** Cardiac Arrythmias, Myocardial Fibrosis, Chagasic Cardiomyopathy, Autonomic Denervation, Norepinephrine

## Abstract

**Background:**

In Chronic Chagas Cardiomyopathy (CCC), studies are needed to identify arrhythmogenic risk factors in patients in which moderate to severe ventricular dysfunction is not present.

**Objective:**

To verify the correlation between frequent ventricular arrhythmias (PVC), left ventricular ejection fraction (LVEF), extension of fibrosis by cardiac magnetic resonance (CMR), and urinary norepinephrine measurement (NOREPI) in CCC with preserved or mildly compromised LVEF.

**Methods:**

The presence of ventricular extrasystoles > 30/h was analyzed on Holter. At CMR, LVEF and quantification of fibrosis mass were evaluated. The dosage of NOREPI was performed using the Muskiet method. The correlation coefficient matrix was calculated to measure the predictive ability of the variables to predict another variable, with p < 0.05 being considered significant.

**Results:**

A total of 59 patients were included. The mean age was 57.9 + 10.94 years. PVC was detected in 28 patients. The fibrosis variable was inversely proportional to LVEF (R of −0.61) and NOREPI (R of −0.68). Also, the variable PVC was inversely proportional to LVEF (R of −0.33) and NOREPI (R of −0.27). On the other hand, LVEF was directly proportional to NOREPI (R of 0.83).

**Conclusion:**

In this sample, in patients with CCC with preserved or slightly reduced LVEF, integrity of the autonomic nervous system is observed in hearts with little fibrosis and higher LVEF despite the presence of traditional risk factors for sudden cardiac death. There is correlation between the levels of NOREPI, LVEF, and myocardial fibrosis, but not with PVC.

## Introduction

Chagas disease (Cd) remains of marked epidemiological importance due to the contingent of infected individuals who are at risk of progression to more severe forms. In Brazil, 1.2 million people are estimated to be infected.^1^ One-third of them have heart disease, two-thirds of these being mild.^2^

Chronic Chagas Cardiomyopathy (CCC) is considered arrhythmogenic due to its potential to cause several fatal types of arrhythmias,^3,4^ especially in more advanced stages of disease (group with increased risk of sudden cardiac death). Although patients at high risk for sudden death can be identified by their risk factors, most sudden deaths occur in patients who were not categorized as high risk.^3^ This apparent paradox hampers the implementation of large-scale preventive measures and justifies research on this group of patients with preserved or mildly reduced ejection fraction.^3,4^

The mechanism behind ventricular arrhythmias in the early stages of CCC may be associated with autonomic denervation, a hallmark of Cd.^4–6^ Studies in the last decade^7–9^ have shown that cardiac autonomic denervation is a common finding in patients with Cd and is caused by neuronal and ganglion inflammation. The destruction and loss of neural cells begin in the acute stage of the disease and continue through the chronic phase, caused by immune or parasitary mechanisms, both acting exclusively or in combination.^10,11^ Autonomic denervation is important in understanding the pathogenesis, as well as the natural history of CCC.

Several authors demonstrated the direct association between sympathetic neural stimulation and the levels of norepinephrine, justifying the use of norepinephrine as a marker of sympathetic activity.^12–14^

In patients with heart failure, sympathetic hyperactivity is persistent and associated with classic adrenergic symptoms, such as tachycardia, sudoresis, diarrhea, and anxiety. However, in Chagas heart disease, these aspects remain controversial, and it has been indicated that the sympathetic system heads towards exhaustion as the cardiac dysfunction progresses.

The common involvement of distal areas of the left ventricle (LV) myocardium, such as the apex and basal inferolateral segments, suggests an acute inflammation leading to myocardial ischemia due to microvascular dysregulation as the pathogenesis of myocardial fibrosis in these patients. Corroborating this, the regulation of abnormal microvascular flow in the presence of chronic myocardial inflammation in CCC was demonstrated by scintigraphy^15–17^ and also magnetic resonance imaging.^18^ These perfusion defects usually precede the appearance of wall motion abnormalities, suggesting that microvascular disorders may develop before the onset of myocardial damage and may be a causative agent of myocardial fibrosis.

The association between CMR findings and arrhythmias in CCC has been studied previously. CMR is currently recommended for patients with severe ventricular arrhythmias in order to quantify myocardial fibrosis and for risk stratification of sudden cardiac death.^19^

Thus, the population with Cd and the potential to develop a cardiac complication is large enough to justify diagnostic strategies that identify patients at increased risk.^20^ Therefore, this study aims to verify the relationship between frequent ventricular arrhythmias, fibrosis extension, ventricular function, regional wall motion abnormalities (RWMA), and urinary norepinephrine dosage in patients with CCC.

## Methods

Patients with CCC aged over 21, with preserved or mildly reduced left ventricular function (EF > 45%) at CMR and who had urinary norepinephrine dosage before the CMR acquisition date were included. The acquisitions were performed between March and December 2010. All patients had a 12-lead electrocardiogram and 24h Holter monitoring before CMR. Only asymptomatic patients away from Chagas endemic zones for more than 20 years and using beta-blockers and angiotensin-converting enzyme inhibitors (ACEI) were included. The exclusion criteria were renal dysfunction (estimated creatinine clearance < 30 mL/min), previous cardiac ablation procedure, diabetes or more than two risk factors for coronary heart disease, atrial fibrillation, treadmill test positive for myocardial ischemia, previous myocardial infarction, any previous myocardial or peripheral revascularization procedure, and standard contraindication to CMR (permanent pacemaker, implanted defibrillator, neurosurgical clip, or cochlear implant).

Holter monitoring was considered positive for frequent ventricular arrhythmia in the presence of premature ventricular contractions (PVCs) > 30/hour, or episodes of non-sustained ventricular tachycardia (NSVT) (defined as three or more consecutive ventricular beats with a duration of less than 30 seconds).^21^

Urinary norepinephrine dosages were performed from 2004 to 2006. All patients were instructed to avoid eating food that contained tyramine (a substance that facilitates the release of norepinephrine from storage sites inside neurons), which could interfere with the norepinephrine concentration, at least 24 hours before and during the urine collecting period. The use of beta-blockers was not suspended during collection. Urine collection was performed on Sundays, starting at 6:00 am, during a period of 24 hours, and all samples were cumulatively stored in two polyethylene bottles with a capacity of one liter each. Every bottle contained 1 ml of 6 M HCl (pH 1.0), with the recommendation to keep the samples at 4° C during the collection period (24h). The method used to determine urinary norepinephrine was based on the proposition by Muskiet et al.^22^

CMR was performed on a 1.5 Tesla GE HDX scanner (Wakeusha, Wisconsin), and two pulse sequences were acquired during end-expiratory breath-hold: the first was cine-CMR (Steady-State Free Precession) in the long-axis and short-axis views for assessing mass, volumes, and LVEF. The most basal cut on the short axis was positioned right after the atrioventricular ring, and all subsequent images were acquired with 8 mm thickness and a 2 mm inter-slice gap, up to the LV apex. The parameters used were field of view (FOV) 400 mm, 224 × 224 matrix, 20-24 lines/segment, temporal resolution < 50 ms, repetition time (TR) = 3.9 ms, echo time (TE) = 1.5 ms, flip angle of 50°, and number of excitations (NEX) of 1. Late gadolinium enhancement was performed three minutes after the injection of 0.3 mmol/kg of gadolinium contrast agent (Dotarem®, Guerbet), using inversion-recovery gradient-echo sequence on the long axis and the short axis (delayed enhancement technique) to search for myocardial fibrosis with the following parameters: FOV 360 mm, matrix 224 × 192, 24 lines/segment, TE = 2.9 ms, flip angle 20°, slice thickness of 8 mm with a spacing of 2 mm and NEX of 2. Two blinded independent readers analyzed all CMR images in a dedicated workstation, using specific software (Report CARD®, version 3.6, GE).

Myocardial fibrosis mass was calculated using a specific software solution through semi-quantitative detection of hyperintense areas in short-axis late enhancement sequences. The investigators were free to edit the limits of the fibrosis area.

The study was approved by the research ethics committee of the Clementino Fraga Filho University Hospital, Universidade Federal do Rio de Janeiro, in compliance with national and international guidelines for research on human beings (Resolution No. 466/2012 of the National Health Council).

### Statistical Analysis

Based on previous studies, known risk factors for electrical instability were used: > 30 PVCs per hour,^21,23^ age,^24^ RWMA,^24,25^ LVEF, and myocardial fibrosis.^25–27^ In addition, urinary norepinephrine dosage was included.

Data normality was verified by the Shapiro-Wilk test as well as with the boxplot and the quartile-quartile plot methods. Normally distributed variables were presented as mean ± standard deviation. Variables that were not normally distributed were presented as median and interquartile ranges.

For arrhythmia analysis, a cutoff value of 720 PVCs in a 24-hour period or the presence of NSVT was used.^21^ RWMA was assessed as the presence or absence by CMR (categorical). To define the cutoff values for LVEF, myocardial fibrosis, urinary norepinephrine level, and age, regression trees were performed using arrhythmia as the outcome.

With the cutoff value already established, a log-linear model was used to measure the dependencies of the variables described above and to confirm the results obtained through the regression tree. The edges of each graph represent the amount of dependence between the variables and outline a number called Cramér's V, which is a digit between 0 and 1 that indicates how strongly two categorical variables are associated. Here is a short statistical explanation: if we want to know if two categorical variables are associated, our first option is the chi-square independence test. A p-value close to zero means that it is very unlikely that the variables will be completely disassociated in a random population. However, this does not mean that the variables are strongly associated. A measure that indicates the strength of the association is Cramér's V.

Then, the correlation coefficient matrix was performed to measure the predictive ability of a continuous variable to predict another variable being analyzed: age, LVEF, fibrosis, arrhythmia, and urinary norepinephrine. The R software was used for data analysis. A p-value < 0.05 was considered significant.

## Results

From 328 outpatients screened, a total of 61 (23 male patients) met the inclusion criteria. Two patients were excluded because they could not undergo the post-contrast phase of CMR (delayed enhancement). One of them due to difficult venous access and the other due to gadolinium atopy.

The main data are shown in Table 1. These are patients with chronic heart disease with normal or mildly reduced ejection fraction. Cardiac fibrosis mass (mean 15.02g) was present in nearly half of the patients and significant ventricular arrhythmias in 47% of them. Urinary norepinephrine levels were variable. Table 2 shows the cutoff values found by linear regression trees.

**Table 1:** General data

Age		
	Mean ± SD	57.9±10.9
**BMI**		
	Mean ± SD	26.1±4.8
**Gender**		
	Female	36
	Male	23
**ECG**		
	ST repolarization abnormalities	33
	LAFB	5
	RBBB	1
	LBBB	1
	RBBB + LAFB	18
	1st-degree AVB	1
**Regional Wall Motion Abnormality**		
	Yes	19
	No	40
**LVEF**		
	45-50%	7
	> 50%	52
	Mean ± SD	66.8 ±11.9
**Fibrosis**		
	Present	27
	Absent	32
	Median (IQ) (g)	0 (0; 10.9)
**24h Holter**		
	Without arrhythmias	12
	Between 1 and 719 PVC	19
	> 720 PVC	28
	Median (IQ)	489.0 (3.0; 1813.5)
**Norepinephrine (nmol/24h)**		
	Median (IQ)	2369.6 (2233.6; 2502.1)
	Without arrhythmia (IQ)	2429.1 (2334.5; 2497.6)
	With arrhythmia (IQ)	2364.1 (2180.1; 2512.3)
	Without fibrosis (IQ)	2437.1 (2342.9; 2759.7)
	With fibrosis (IQ)	2327.4 (1461.1; 2429.1)

BMI: body mass index; ECG: electrocardiogram; LAFB: left anterior fascicular block; RBBB: right bundle branch block; LBBB: left bundle branch block; AVB: atrioventricular block; LVEF: left ventricular ejection fraction; PVC: premature ventricular contraction; IQ: interquartile; SD: standard deviation.

**Table 2:** Results of the linear regression tree for the cutoff points for the log-linear model

LVEF (n)	RWMA	Arrythmia (n)	Fibrosis (n)	Norepinephrine (n)	Age (n)
≤57% (13)	No (41)	No (31)	≤10.56% (44)	≤2218.97 nmol/24h (15)	≤54 years (20)
>57% (46)	Yes (18)	Yes (28)	>10.56% (15)	>2218.97 nmol/24h (44)	>54 years (39)

LVEF: left ventricular ejection fraction; RWMA: Regional Wall Motion Abnormality.

A multivariate analysis using the Loglinear model was used to verify the pattern of interaction (dependence) of the variables shown in Figure 1.

**Figure 1:** Log-Linear Model. Edge weights correspond to the Cramér's V statistic (measure of dependence between discrete variables).

The variables fibrosis, LVEF, and norepinephrine are observed to have a direct pattern of interaction (dependence) with each other, with a high power of association (fibrosis and norepinephrine 0.64, LVEF and norepinephrine 0.63, and fibrosis and LVEF 0.53). These interactions are of second order. Fibrosis is associated with arrhythmia depending on RWMA through a third-order interaction, that is, the three variables must be present. It is also noted that there is no direct interaction between arrhythmia and norepinephrine.

A correlation coefficient matrix was performed, where R demonstrates, in percentage values, how much the five variables are correlated with each other (Figure 2). Variables with the greatest relation (directly or inversely proportional) have the most oval circumference. Asterisks represent the level of significance according to the p-value (*** p < 0.001, ** p < 0.1, * p < 0.05). Fibrosis was inversely proportional to LVEF (R = −0.61) and urinary norepinephrine (R = −0.68). The variable arrhythmia was shown to have an inverse relationship with LVEF (R = −0.33) and urinary norepinephrine (R = −0.27). LVEF was directly proportional to norepinephrine (R = 0.83).

**Figure 2:** Correlation Coefficient Matrix (values in R). The more oval the better the correlation. Asterisks represent the significance according to the p-value. (*** p < 0.001, ** p < 0.1, * p < 0.05).

## Discussion

This is the first study on CCC with mild fibrosis and preserved or mildly reduced ejection fraction to show significant dependence between traditional risk factors. More fibrosis was associated with lower norepinephrine levels. Higher LVEF was associated with higher norepinephrine levels, as shown in the correlation matrix (-0.68 and 0.83, respectively). It was also demonstrated that the presence of arrhythmia was not associated with the other variables.

This is a novel publication as it demonstrates for the first time that risk factors for sudden death may already be present, such as sympathetic denervation, myocardial fibrosis, and frequent ventricular arrhythmias, even in patients with preserved or mildly reduced ejection fraction. Furthermore, this finding is of great importance because most Cd patients have normal or mildly reduced ventricular function.

Dependence of higher norepinephrine dosages on higher LVEF in the correlation matrix was also demonstrated by Iosa et al.^28^ They demonstrated the inverse relationship between cardiac dysfunction in CCC and norepinephrine levels. In the late phases of Chagas cardioneuropathy, plasma norepinephrine levels remained normal, unlike patients with non-Chagas heart failure, who had higher norepinephrine levels the greater the ventricular dysfunction.

Cd causes autonomous nervous system (ANS) lesions during the acute and chronic phases of the disease. This justifies the correlation between norepinephrine levels, fibrosis, and LVEF observed in this study. These patients seem to have sympathetic denervation caused by progressive neuronal destruction, reflected by the inverse relationship between norepinephrine levels and myocardial fibrosis, as shown in the correlation matrix (Figure 2).

Catecholamine levels are known to vary greatly throughout the circadian cycle, during venipuncture,^29^ or even if the patient is hospitalized.^30^ Most of the published norepinephrine test results are based on plasma samples and few studies have been conducted in Cd. Ross et al.^31^ demonstrated that 24-hour urine norepinephrine dosages reduce false-negative results in patients with pheochromocytoma.

We found only one clinical study using urinary norepinephrine in Cd. Cunha et al.^32^ evaluated the involvement of the ANS in the pathogenesis of CCC. They observed decreased urinary norepinephrine levels in CCC with ventricular dysfunction and, conversely, normal or even increased levels in patients with the indeterminate form of Cd.

As previously demonstrated by our group,^25^ fibrosis is inversely associated with LVEF. In the current study, fibrosis, RWMA, and norepinephrine have second-order interactions (direct dependence) with LVEF, observed by the Loglinear method. Together or individually, they cause ventricular remodeling, partially justifying the Myerburg model.^33^ In the previous study, there was a third-order interaction (the three variables must be present) between fibrosis, arrhythmia, and LVEF, which was not maintained in the current study. This can probably be explained in two different ways: firstly, since sympathetic denervation morphologically occurs before the onset of fibrosis^9,34^ this can balance the dependence of the arrhythmia, losing explanatory power; secondly, design, as in the first study the log-linear only considered patients with LVEF above 50% by CMR, that is, seven patients with mild dysfunction (LVEF between 45-50%) were removed, of which six had frequent arrhythmias. This could justify the loss of interaction between the variables.

In our study, the mean myocardial fibrosis weight was 15.02g (Table 1), and the value of 10.56% (10.01g) determined the presence of frequent arrhythmias according to the regression tree. Recently, Senra et al.^26^ observed in a retrospective prognostic study that myocardial fibrosis is an independent risk factor (for death, implantable defibrillator triggering, and heart transplantation) in CCC and that each additional gram of fibrosis would increase by 3.1 % the risk of a hard event. They detected an average weight of fibrosis (15.2g or 13.5%) very close to that found in our study. Also, based on a ROC curve analysis, they determined that the cutoff value of 12.3g is a predictor of a major event. In the same year, Volpe et al.^27^ in another retrospective prognostic study of about 3 years of follow-up in patients with CCC, detected 10.4g of fibrosis (9.2% of LV mass) and there were 11 deaths, of which 10 had detectable fibrosis.

Gadioli et al.^9^ found a proportional relation between arrhythmia severity (SVT and NSVT) and the extension of sympathetic denervation, similar to what was shown in this study, as seen by the presence of arrhythmia with lower levels of norepinephrine, denoting greater denervation. However, they did not find a relation between fibrosis detected by 99mTc-Sestamibi and ventricular arrhythmia, which occurred in this study. This can be explained by the higher spatial resolution of CMR compared to myocardial scintigraphy to detect myocardial fibrosis.

This study has some limitations. The definition of frequent ventricular arrhythmia in the present study may be questionable from a clinical point of view, but not functional in terms of neurogenic modulation. However, when using a low-risk Cd population (LVEF > 45% and mean age 57.9 years) and focusing on the anatomopathological basis of the arrhythmogenic substrate of fibrosis and not on clinical instability due to malignant arrhythmia, it is confirmed that the arrhythmogenic substrate is already present in this population. Likewise, it is evident in this study that the predominant pathogenic mechanism in this population is neurogenic and not cardiac, a fact that has also been published by other authors.^8,35^

Although we cannot definitively rule out the diagnosis of coronary heart disease as an important confounding factor, clinical data and the pattern of fibrosis allow us to infer the absence of functionally significant obstructive coronary disease. Furthermore, this group of patients did not have indications for coronary angiography.^36^

The time interval between urinary norepinephrine dosage and CMR acquisition (about 6 years) may seem long. However, it is important to remember that progression during the early stages of cardiac involvement is very slow, about 1.48 cases/100 patient-years.^37^ So, at most, six patients may have changed groups during the study.^38^

The patients were previously using beta-blockers and angiotensin-converting enzyme inhibitors (minimum of 6 months). These drugs were used according to recommendations for Cd^39^ with cardiac involvement (stages A and B1). The decision for pharmacological treatment was within the clinical context and not intended to control or treat arrhythmias. Therefore, even if these drugs modulate the neurohormonal response, they would not be a limitation of the study since the adrenergic load was established during the chronic use of these drugs.

The Loglinear model shows that arrhythmia quantification is not related to LVEF, fibrosis, and/or norepinephrine dosage, and fibrosis is related to urinary norepinephrine and LVEF with similar explanatory power to each other (Cramér's V statistic of 0.53, 0.63, and 0.64). Indeed, there are data in the Cd literature that the presence of ventricular arrhythmia and/or right bundle branch block (RBBB) are not independent prognostic markers for all-cause mortality. However, they are markers of cardiac involvement.^21^ The sudden death mechanism in Cd is due to ventricular tachycardia or fibrillation, not necessarily preceded by complex arrhythmias, which are more intensively associated with LV dysfunction.^4^

## Conclusions

In patients with CCC with preserved or mildly reduced ejection fraction, ANS integrity is observed in hearts with little fibrosis and higher LVEF despite the presence of traditional risk factors for sudden cardiac death. There is a correlation between the levels of urinary norepinephrine, LVEF, and myocardial fibrosis, but not with the presence of frequent ventricular arrhythmias.
